# Dysregulated RNA polyadenylation contributes to metabolic impairment in non-alcoholic fatty liver disease

**DOI:** 10.1093/nar/gkac165

**Published:** 2022-03-16

**Authors:** Andrew M Jobbins, Nejc Haberman, Natalia Artigas, Christopher Amourda, Helen A B Paterson, Sijia Yu, Samuel J I Blackford, Alex Montoya, Marian Dore, Yi-Fang Wang, Alessandro Sardini, Inês Cebola, Johannes Zuber, Sheikh Tamir Rashid, Boris Lenhard, Santiago Vernia

**Affiliations:** MRC London Institute of Medical Sciences, Du Cane Road, London W12 0NN, UK; Institute of Clinical Sciences, Imperial College London, Hammersmith Hospital Campus, Du Cane Road, London W12 0NN, UK; MRC London Institute of Medical Sciences, Du Cane Road, London W12 0NN, UK; Institute of Clinical Sciences, Imperial College London, Hammersmith Hospital Campus, Du Cane Road, London W12 0NN, UK; MRC London Institute of Medical Sciences, Du Cane Road, London W12 0NN, UK; Institute of Clinical Sciences, Imperial College London, Hammersmith Hospital Campus, Du Cane Road, London W12 0NN, UK; MRC London Institute of Medical Sciences, Du Cane Road, London W12 0NN, UK; Institute of Clinical Sciences, Imperial College London, Hammersmith Hospital Campus, Du Cane Road, London W12 0NN, UK; MRC London Institute of Medical Sciences, Du Cane Road, London W12 0NN, UK; Institute of Clinical Sciences, Imperial College London, Hammersmith Hospital Campus, Du Cane Road, London W12 0NN, UK; MRC London Institute of Medical Sciences, Du Cane Road, London W12 0NN, UK; Institute of Clinical Sciences, Imperial College London, Hammersmith Hospital Campus, Du Cane Road, London W12 0NN, UK; Department of Metabolism, Digestion & Reproduction, Imperial College London, London W12 0NN, UK; MRC London Institute of Medical Sciences, Du Cane Road, London W12 0NN, UK; Institute of Clinical Sciences, Imperial College London, Hammersmith Hospital Campus, Du Cane Road, London W12 0NN, UK; MRC London Institute of Medical Sciences, Du Cane Road, London W12 0NN, UK; Institute of Clinical Sciences, Imperial College London, Hammersmith Hospital Campus, Du Cane Road, London W12 0NN, UK; MRC London Institute of Medical Sciences, Du Cane Road, London W12 0NN, UK; Institute of Clinical Sciences, Imperial College London, Hammersmith Hospital Campus, Du Cane Road, London W12 0NN, UK; MRC London Institute of Medical Sciences, Du Cane Road, London W12 0NN, UK; Institute of Clinical Sciences, Imperial College London, Hammersmith Hospital Campus, Du Cane Road, London W12 0NN, UK; Section of Genetics and Genomics, Department of Metabolism, Digestion & Reproduction, Imperial College London, London W12 0NN, UK; Research Institute of Molecular Pathology (IMP), Vienna BioCenter (VBC), 1030 Vienna, Austria; Department of Metabolism, Digestion & Reproduction, Imperial College London, London W12 0NN, UK; MRC London Institute of Medical Sciences, Du Cane Road, London W12 0NN, UK; Institute of Clinical Sciences, Imperial College London, Hammersmith Hospital Campus, Du Cane Road, London W12 0NN, UK; MRC London Institute of Medical Sciences, Du Cane Road, London W12 0NN, UK; Institute of Clinical Sciences, Imperial College London, Hammersmith Hospital Campus, Du Cane Road, London W12 0NN, UK

## Abstract

Pre-mRNA processing is an essential mechanism for the generation of mature mRNA and the regulation of gene expression in eukaryotic cells. While defects in pre-mRNA processing have been implicated in a number of diseases their involvement in metabolic pathologies is still unclear. Here, we show that both alternative splicing and alternative polyadenylation, two major steps in pre-mRNA processing, are significantly altered in non-alcoholic fatty liver disease (NAFLD). Moreover, we find that Serine and Arginine Rich Splicing Factor 10 (*SRSF10*) binding is enriched adjacent to consensus polyadenylation motifs and its expression is significantly decreased in NAFLD, suggesting a role mediating pre-mRNA dysregulation in this condition. Consistently, inactivation of SRSF10 in mouse and human hepatocytes *in vitro*, and in mouse liver *in vivo*, was found to dysregulate polyadenylation of key metabolic genes such as peroxisome proliferator-activated receptor alpha (*PPARA)* and exacerbate diet-induced metabolic dysfunction. Collectively our work implicates dysregulated pre-mRNA polyadenylation in obesity-induced liver disease and uncovers a novel role for SRSF10 in this process.

## INTRODUCTION

Overnutrition and obesity are strongly linked to the development of non-alcoholic fatty liver disease (NAFLD) and further progression to its more severe form, non-alcoholic steatohepatitis (NASH). These metabolic pathologies are becoming an important health threat, especially in western society due to the prevalence of high calorie diets (1,2). NAFLD and NASH are associated with metabolic syndrome and a progressive increase in insulin resistance, both of which are risk factors for type 2 diabetes, cirrhosis and hepatocellular carcinoma (HCC). There are currently no effective therapeutic interventions for NAFLD or NASH. Consequently, there is a large interest in elucidating mechanisms underlying the onset and progression of obesity-induced fatty liver disease.

The maturation of pre-mRNA is a central step in eukaryotic gene expression; acting as a key mechanism for controlling expression levels and proteomic diversity (3–5). Over 95% of human multi-exon genes undergo alternative splicing (AS) and >50% have alternative polyadenylation (APA) sites (6,7). The outputs from these two processes can be exceptionally diverse, from changes in the coding sequence itself to alterations in the untranslated regions which can modulate nuclear export, RNA stability, protein localization and translation initiation and termination (8–11). Evidence shows that directed specific regulation of both pre-mRNA splicing and polyadenylation is essential for the maintenance and establishment of developmental, tissue, temporal and inter-species differences in gene expression (5,12–17). The regulation of both processes relies on *cis*-acting RNA binding proteins and while the overlap of factors which affect both processes was initially limited to U1 snRNP associated factors, involved in 5′ splice site recognition and telescripting, it has recently been expanded to include a number of additional factors (18–24). Research has even suggested that the mechanism for regulation by RNA binding proteins between the two processes is conserved (25). Given its central role in gene expression, RNA processing is a tightly controlled process. However, its dynamism, necessary for increased proteomic diversity, means that it is vulnerable to errors with an increasing number of diseases implicating RNA processing in their development (26–28).

While it has been suggested that a number of RNA-binding proteins are dysregulated in human insulin-resistant liver samples (29–32) the contribution of defects in RNA processing to obesity-induced liver disease has not been systematically addressed. The SR and hnRNP factors are two classical RNA binding protein families involved in RNA processing. SR proteins were originally described as activators of splicing, able to stimulate splicing in S100 extracts (33–36), whilst hnRNP proteins were repressors, able to compete with SR proteins to prevent splice site usage (37,38). However, as research has progressed, location and posttranslational modification dependent effects have been found (39–46). Furthermore, the non-splicing functions of SR protein have expanded to include transcription, nuclear export and polyadenylation suggesting that they are able to regulate all steps in the maturation of mRNA (19).

Here, we find that NAFLD is associated with a dysregulation of pre-mRNA processing at the alternative splicing and polyadenylation level. Analysis of crosslinking and immunoprecipitation (CLIP) data, proximity labelling interactome and further functional studies identify the multi-functional Serine and Arginine Rich Splicing Factor 10 (SRSF10), as a key factor in this process in mouse and human hepatocytes. Mechanistically, our work uncovers a novel role for SRSF10 in repressing cryptic intronic polyadenylation signals to maintain the expression of key metabolic genes, preventing the development of obesity-induced liver pathology.

## MATERIALS AND METHODS

### Human samples and RNA-seq analysis

Previously published datasets of early changes in the initiation of NAFLD (control: *n* = 10 and NAFLD: *n* = 51) were obtained from GSE135251 (47).

Reads were aligned to Ensemble mouse genome (GRCm38) or UCSC human genome (hg38) using STAR (2.7.7a; (48)) with argument ‘-quantMode TranscriptomeSAM GeneCounts’. Gene-based read counts were performed by STAR as well. Normalization and differential expression analysis were performed using DEseq2 bioconductor package(49). Alternative splicing was analysed with rMATs (4.1.1; (50)). The splice sites were kept for data visualization if FDR <0.05 and passed the following thresholds: for Alt3 and Alt5: ≥10 actual reads mapping to the sum of all exon:exon junctions (EEJs) involved in a specific event. For RI: (i) skipping junction counts (SJC) ≥10, or (ii) inclusion junction counts (IJC) ≥10 in one of the two IJC and ≥5 to the other IJC. For SE and MXE: (i) SJC ≥10 or (ii) IJC ≥10 in one of the two IJC and ≥5 to the other IJC. KEGG gene set analysis was done in http://www.gsea-msigdb.org/gsea/msigdb/annotate.jsp. Alternative polyadenylation analysis was performed using QAPA [v1.3.1; (51)]. Gene expression data downloaded from GSE73299 (52) was used to determine PPARα-regulated genes in the liver. For the differential expression analysis statistical *t*-test was used together with log_2_ fold change measurements by using custom R-script. For the comparison of differentially expressed genes of SRSF10-KD and PPARα-KO samples same log_2_ fold change values were used with the threshold of *P*-value lower than 0.05. For the final visualization of log_2_ fold change values a Pearson correlation was used and visualized with linear regression line in R (version 4.1.2) using ggplot2 package.

### RNA isolation and PCR analysis

RNA was isolated with TRIzol (Thermo Fisher Scientific) following the manufacturer instructions. For RNA sequencing, after homogenization with TRIzol, RNA was extracted with a RNeasy kit column (Qiagen), including DNase I treatment using standard protocols. List of primers and probes is provided in Supplementary Table S2.

### RNA sequencing

RNA samples were QCed using the Agilent 2100 Bioanalyser RNA 6000 Nano kit and libraries were made using the NEBNext Poly(A) mRNA Magnetic Isolation Module and NEBNext^®^ Ultra^™^ II Directional RNA Library Prep Kit for Illumina kits. Subsequent libraries were QCed using the Agilent 2100 Bioanalyser High Sensitivity Assay and the Qubit fluorometer. Pooled libraries were run on a Paired End 75 + 8 bp dual index High Output NextSeq 550 run to generate 50 million reads per sample.

### Consensus PolyA motif enrichment relative to SR binding sites

To explore the SRSF10 and its link to polyadenylation we used SR PAR-CLIP dataset (GEO access: GSE71096) from human HeLa cells. This dataset contains pre-processed binding sites from several SR proteins (SRSF1, SRSF3, SRSF7, SRSF9, SRSF10) aligned to hg19 genome annotation. For each SR protein binding start site, we extracted genomic sequences in 100 nts flanking region by using *bedtools getfasta* function and custom Python scripts. Next, we counted consensus PolyA motifs (AATAAA, ATTAAA, AAATAA, ATAAAA and ATAAAT) in the surrounding region, where we summed the matched motifs in the region and normalized them by the number of binding sites for each SR protein separately. For the final visualization of motif enrichment graphs, we used R (version 4.0.3) together with the following Bioconductor packages: *ggplot2*, *smoother* and *cowplot*.

### Differential expression analysis of transposable elements (SINES)

In order to investigate expression changes in SINE elements from RNA-seq samples, we ran Bioconductor tool for differentially expression analysis DESseq2 (version 1.32) with the FDR <0.05 thresholds. The SINEs were extracted from UCSC Transposable Elements tables for hg38 and mm10 annotation. For each SINE element count tables were created by using bedtools (version 2.3). This was done for both sets of samples: mouse *Srsf10-kd* versus *Control* samples and randomly selected (12) human NAFLD vs Control (eight samples) samples to reduce the computational power of differentially expression analysis. Log_2_-fold-change was used for the visualization of expression changes between the conditions and controls.

### Differential expression analysis of IPAs

IPA candidates were first identified by using IPAFinder tool (https://github.com/ZhaozzReal/IPAFinder) from paired-end RNA-seq samples of *Srsf10-kd* against *Ctrl* from mouse liver tissue. For the IPA discovery we used recommended annotation provided by IPAFinder package based on RefSeq-mm10 annotation. The identified IPAs were then used for differential expression analyses by running un-paired student t.test and ‘EnhancedVolcano’ R package for the final visualization.

### Mice

C57BL/6J mice were obtained from Charles River and housed in pathogen-free barrier facilities under 12-h light/dark cycles at 22°C.

Glucose, insulin, and pyruvate tolerance tests were performed following 16-h fasts (4 h for insulin) followed by intraperitoneal (IP) injection of mice with glucose (2 g/kg) or insulin (0.5 U/kg) and glucose measured at appropriate timepoints. Biopsies were flash frozen in liquid nitrogen and kept at −80ºC. Sections for histology were fixed in 10% formalin and subsequently embedded in paraffin for hematoxylin & eosin staining. All *in vivo* work was approved by the animal welfare and ethical review board at Imperial College London and in accordance with the United Kingdom Animals (Scientific Procedures) Act (1986).

### Protein analysis

Tissue was homogenized in Triton Lysis Buffer (12.5 mM HEPES pH 7.4, 50 mM NaCl, 500 μM EDTA, 5% glycerol, 0.5% Triton X-100, 50 mM sodium vanadate, 50 mM PMSF, 5 mM aprotinin, 5 mM, Leupeptin) using a TissueLyser II Homogenizer (Qiagen) before undergoing centrifugation at 10 000 rpm for 10 min at 4°C. The supernatant containing the protein lysate was transferred to a new tube and protein quantified with Pierce BCA Protein Assay Kit (Thermo Fisher Scientific) and analysed by western blot by incubating with anti-SRSF10 (Cell Signalling), anti-SRSF1 (Thermofisher), anti-SRSF5 (Atlas), anti-PPARα (Abcam), anti-Lamin (Abcam), anti-actin (Santa Cruz) and anti-Tubulin (Santa Cruz sc-5286) primary antibodies and imaged with an Odyssey infra-red scanner (LICOR).

### RNA crosslinking and immunoprecipitation

100mg liver powder, on dry ice, was cross linked with UV (254 nm, 400 mJ/cm^2^) three times. AG dynabeads were washed twice in lysis buffer (50 mM Tris−HCl (7.4), 100 mM NaCl, 1% Igepal CA-630, 0.1% SDS, 0.5% sodium deoxcholate) and incubated with 10 μg antibody rotating at 4°C for 1 h then washed once in high salt buffer (50 mM Tris–HCl (7.4), 1 M NaCl, 1 mM EDTA, 1% Igepal CA-630, 0.1% SDS, 0.5% sodium deoxcholate) and twice in lysis buffer. Liver powder was resuspended in 1 ml lysis buffer (+Proteinase inhibitors & RNase inhibitors) and lysed using a TissueLyser II Homogenizer (Qiagen). 16 mg of protein was incubated at 37°C for 3 min with 10μl 1/1000 RNaseI (in lysis buffer) and 2 μl Turbo DNase then 3 min on ice. Samples were span at 18 000g for 10 min and the supernatant mixed with antibody bound beads and incubated at 4°C rotating for 1 h. Beads were washed twice with high salt buffer. 1/10 of beads were kept to assess immunoprecipitation efficiency and the rest treated with 50 μg PK to release RNA. RNA was purified using RNeasy kit column (Qiagen) and quantified using a nanodrop.

### Plasmids and viral vectors

*AAV-CBA-mirE/shRNA* vector was generated by gene synthesis containing chicken β–actin (CBA) promoter driving the expression of GFP and the miR-30 backbone as previously optimized (53), including XhoI/EcoRI sites for simplified cloning of mirE/shRNA sequences. Additional HindIII/BamHI and BsrGI/NotI were included surrounding GFP to enable efficient subcloning of other transgenes. This sequence was surrounded by serotype 2 specific inverted terminal repeats (ITR2) to enable single stranded AAV production. Sequences were confirmed by Sanger DNA sequencing and are available upon request. *In vitro* experiments with mirE/shRNAs were performed with LT3GEPIR vector (53).

AAV-Luciferase-intron or AAV-Luciferase-control vectors were obtained by subcloning the optimized Luciferase-intron and Luciferase-Control transgenes (54) into an ITR2 vector containing thyroxine binding globulin (TGB) promoter for efficient production of single stranded AAV viruses.

### Production and injection of adeno-associated viruses

Recombinant AAV serotype 2/8 (AAV2/8) were prepared and amplified as previously described (55,56). Male mice (6 weeks old) were injected by tail vein injection with 2.5 × 10^11^ to 5 × 10^11^ genome copies per mouse. HFD was begun 4 weeks post injection.

### *In vivo* splicing reporter

Six-week-old C57B6J male mice were injected via tail vein with 5 × 10^11^ genome copies or AAV-Luciferase-intron or AAV-Luciferase-control. Mice were fed with high-fat or a chow control diet for 12 weeks. After this time, d-luciferin was dissolved in water at 30mg/ml and mice were injected i.p. with 0.15mg/g body weight before being anesthetized with isoflurane. Mice were kept are 37°C on the stage of the instrument. Ten minutes after D-luciferin injection mice were imaged in an IVIS Spectrum (Perkin Elmer).

Images were analysed with the Living Image software (Caliper Life Science) by quantifying the signal flux from the liver region as average radiance (photon/s/cm^2^/sr).

### 3′RACE

cDNA was reverse transcribed from 4 mg of RNA using a oligo dT primer with a 3′ adapter sequence as previously described (57). Subsequent PCR reactions were performed using a reverse primer complementary to the adapter and gene specific forward primers. Sequencing was performed following a subsequent nested PCR reaction.

### Proximity labelling assay—sample processing

Plasmids expressing BirA were purchased from addgene (#74224 & #74223). Mouse *Srsf10* open reading frame was cloned into BamHI/EcoRI sites at the N terminus of the BirA tag. 30 μg plasmid, SRSF10-BirA or BirA, were transfected into HeLa cells. The following day, cells were changed to media supplemented with 50 μM biotin (Sigma-Aldrich) and incubated overnight. Cells were washed twice in phosphate-buffered saline (PBS) and harvested. Fusion construct expression and biotinylation were confirmed by western blot. Biotinylated proteins were captured from 500 mg of protein using streptavidin coated dynabeads (Invitrogen).

Samples were processed using an on-bead digestion procedure. Briefly, beads were suspended in 4 M urea in 20 mM HEPES (pH 8.0) and transferred to fresh lo-bind tubes. Samples were placed on a magnetic rack and clarified supernatant from each tube used to wash original tubes for recovery of residual beads. Samples were digested for 5 h with 1.5 μg of LysC/Trypsin (Promega, V5071) at 37°C with shaking. Bead slurry was further diluted to approximately 1 M urea by addition of 20 mM HEPES (pH 8.0) containing 2 mM DTT. Samples were further incubated at 37°C over-night. Post digestion, clarified digest solutions were recovered with use of the magnetic rack. Beads were sequentially washed with 20 mM HEPES twice, with clarified supernatants pooled with relevant solutions. Samples were acidified with 1% trifluoroacetic acid (TFA) to a final concentration of 0.1% and protein digests were desalted using Glygen C18 spin tips (Glygen Corp, TT2C18.96). Tryptic peptides were eluted with 60% acetonitrile, 0.1% formic acid (FA). Eluents were dried by vacuum centrifugation.

### Proximity labelling—liquid chromatography–tandem mass spectrometry (LC–MS/MS)

Dried tryptic digests were re-dissolved in 0.1% TFA by shaking (1200 rpm) for 30min at room temperature and then pulse sonicated on an ultrasonic water bath for 5min twice, followed by centrifugation (13 000 rpm, 5°C) for 10 min. LC–MS/MS analysis was performed using an Ultimate 3000 RSLC nano liquid chromatography system (Thermo Scientific) coupled to a Q-Exactive mass spectrometer (Thermo Scientific) via an EASY spray source (Thermo Scientific). For LC–MS/MS analysis re-dissolved protein digests were injected and loaded onto a trap column (Acclaim PepMap 100 C18, 100 μm × 2 cm) for desalting and concentration at 8 μl/min in 2% acetonitrile, 0.1% TFA. Final on-column digest concentration was 600 ng per injection. Peptides were then eluted on-line to an analytical column (Acclaim Pepmap RSLC C18, 75 μm × 75 cm) at a flow rate of 200 nl/min. Peptides were separated using a 120 min gradient, 4–25% of buffer B for 90 min followed by 25–45% buffer B for another 30 min (composition of buffer B–80/20%, acetonitrile/ H_2_O + 0.1% FA) and subsequent column conditioning and equilibration. Eluted peptides were analysed by the mass spectrometer operating in positive polarity using a data-dependent acquisition mode. Ions for fragmentation were determined from an initial MS1 survey scan at 70 000 resolution, followed by HCD (Higher Energy Collision Induced Dissociation) of the top 12 most abundant ions at 175 000 resolution. MS1 and MS2 scan AGC targets were set to 3e6 and 5e4 for maximum injection times of 50 and 50 ms, respectively. A survey scan *m*/*z* range of 400–1800 was used, normalized collision energy set to 27%, charge exclusion enabled with unassigned and +1 charge states rejected and a minimal AGC target of 1e3. Dynamic exclusion was set to 30 s.

Data was processed using the MaxQuant software platform (v1.6.2.3), with database searches carried out by the in-built Andromeda search engine against the Uniprot *Homo sapiens* database (version 20180104, number of entries: 172 263) concatenated with mouse SRSF10 sequence. A reverse decoy search approach was used at a 1% false discovery rate (FDR) for both peptide spectrum matches and protein groups. Search parameters included: maximum missed cleavages set to 2, variable modifications of methionine oxidation, protein N-terminal acetylation, lysine biotinylation and protein N-terminal biotinylation. Label-free quantification was enabled with an LFQ minimum ratio count of 2. ‘Match between runs’ function was used with match and alignment thresholds of 1 and 20 min, respectively. Hits were shortlisted to the top highest confidence interactors using >4 razor and unique peptides, >20% sequence coverage, <0.05 *P* value (Student's *t*-test) versus free BirA and a log_2_ fold change >1.

### Quantification of liver triglycerides

50–200 mg liver was weighed, added to 350 μl ethanolic KOH (2 ethanol (100%):1 KOH (30%)) and incubated overnight at 50°C. Samples were vortexed and 650 μl of ethanol (50%) was added followed by centrifugation at full speed for 5 min. 900 μl of the of the supernatant was mixed with 300 μl ethanol (50%). 200 μl of the samples were mixed with 215 μl of 1 M MgCl_2_ and incubated on ice for 10 min and subsequently centrifuged at full speed for 5 min. 10 μl of the supernatant was assayed for glycerol content using Sigma free glycerol reagent (F6428).

### Mouse primary hepatocyte isolation

Mouse primary hepatocytes were isolated by first perfusing livers with liver perfusion media (Hanks buffered saline solution with 0.4g/l KCl, 1 g/l glucose, 2.1 g/l NaHCO_3_ and 0.2 g/l EDTA) and liver digest media containing DMEM (1 g/l gucose, +Glutamax) with 15 mM HEPES (pH 7.4), 1% PenStrep and 50 mg/l Collagenase (type 4). Cells were seeded for 3 h on collagen coated plates (1 h rat tail collagen I) in DMEM (4.5 g/l glucose, 10% FBS, 0.2% BSA, 2 mM sodium pyruvate, 2 mM glutamine, 1 μM dexamethasone, 100 nM insulin and 1% Pen/Strep). siRNAs were purchased from Dharmacon (smartpool) and transfected at 100 nM with RNAimax (Invitrogen) in Opti-MEM overnight.

### Human iPSC derived hepatocytes

Human induced pluripotent stem cell line (iPSC): CGT-RCiB-10 (Cell & Gene Therapy Catapult, London, UK). iPSCs were maintained on Vitronectin XF (STEMCELL Technologies) coated Corning Costar TC‐treated six‐well plates (Sigma–Aldrich) in Essential 8 Medium (Thermo Fisher Scientific) and passaged every 4 days using Gentle Cell Dissociation Reagent (STEMCELL Technologies).

Hepatocyte differentiation was carried out as previously described (58) in Essential 6 Medium (Thermo Fisher Scientific; days 1–2), RPMI‐1640 Medium (Sigma–Aldrich; days 3–8) and HepatoZYME‐SFM (Thermo Fisher Scientific; day 9 onward) within TC-treated 182 cm^2^ flasks (VWR). The following growth factors and small molecules were supplemented into the media for hepatocyte differentiation: 3 μM CHIR9901 [Day 1] (Sigma–Aldrich), 10 ng/ml BMP4 [day 1–2] (R&D Systems), 10 μM LY29004 [day 1–2] (Promega, Madison, WI), 80 ng/ml FGF2 [day 1–3] (R&D Systems), 100 ng/ml [day 1–3] and 50 ng/ml [day 4–8] Activin A (Qkine), 10 ng/ml OSM [day 9 onwards] (R&D Systems) and 50 ng/ml HGF [day 9 onwards] (PeproTech). After 21 days iPSC-derived hepatocytes were dissociated into a single‐cell suspension using TrypLE Express Enzyme (10×), no phenol red (Thermo Fisher Scientific) and seeded into multi-well plates coated with type-1 collagen from rat tail (Sigma–Aldrich).

Third generation lentivirus was generated in HEK-293T cells and purified by high-speed centrifugation. Virus was resuspended in media supplemented with polybrene and added to iPSC derived hepatocytes.

### Statistical analysis

Differences between groups were examined for statistical significance using Student's *t*-tests, Mann–Whitney or one/two-way ANOVAs where indicated.

## RESULTS

### Alternative polyadenylation is dysregulated in NAFLD human samples

Defects in pre-mRNA splicing are emerging as an important factor underlying a number of human pathologies including neurodegeneration and cancer(26,27). In order to investigate the contribution of pre-mRNA processing to the early stages of human NAFLD, we analysed exon-usage and gene expression changes in a cohort of NAFLD patients(47) with rMATS(50) and DESeq2(49) respectively.

**Figure 1.**
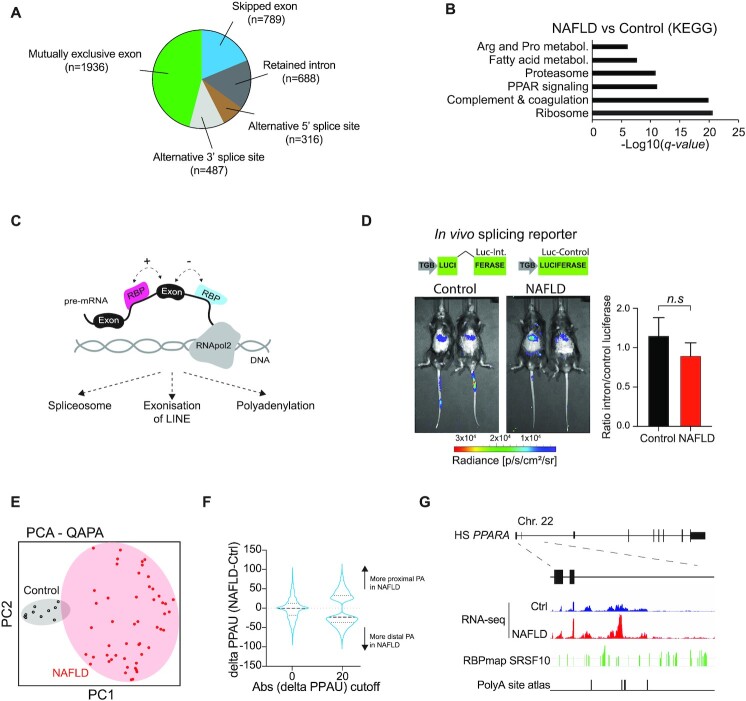
Non-alcoholic liver disease is associated with dysregulation of mRNA polyadenylation. (**A**) Distribution of significant (FDR < 0.05) AS events as identified in human NAFLD data set. (**B**) KEGG gene set analysis of genes showing differential intron retention in NAFLD. (**C**) Cartoon depicting a hypothetical model for mRNA processing pathways involved in intron retention. (**D**) Bioluminescence analysis of mice expressing a liver-specific *in vivo* splicing reporter (*n* = 4–5). (**E**) Principal component analysis plot of NAFLD versus Ctrl polyadenylation analysis (QAPA). (**F**) Distribution of increased or decreased proximal polyadenylation usage with two different thresholds, >0 or >20 (dPPAU). (**G**) RNAseq tracks in the intron 2 of human *PPARA* gene in liver from NAFLD and control samples. Predicted SRSF10 binding sites are marked with green lines. Bottom track shows validated 3′ RNA sequencing data indicating potential intronic polyadenylation sites.

The analysis showed broad exon-usage changes in the livers of individuals with early stage NAFLD, which included mutually exclusive exons (*n* = 1936), skipped exons (*n* = 789), retained introns (*n* = 688), alternative 3′ splice sites (*n* = 487) and alternative 5′ splice sites (*n* = 316) (Figure 1A). Gene ontology analysis of genes with significant changes in intron retention showed an enrichment in genes involved in ribosome function, Peroxisome Proliferator Activated Receptor (PPAR) signalling and fatty acid metabolism (Figure 1B). Since intron retention could be associated with defects in RNA-processing and results in the misexpression of key metabolic genes we decided to investigate the molecular mechanisms underlying these changes. We hypothesized that increases in intronic reads could be caused by general defects in the core splicing machinery, by the exonization of intronic sequences such as endogenous retro-transposable elements and/or by changes in intronic and alternative polyadenylation (Figure 1C).

To our knowledge, no *in vivo* liver splicing reporters have been described in mammals. To investigate the activity of mRNA splicing machinery *in vivo*, we first generated a bioluminescent liver-specific real-time splicing reporter. Briefly, we produced adeno-associated viruses (AAVs) expressing luciferase with (Luc-intron) or without (Luc-control) an artificial intron under the human thyroxine binding globulin (TGB) promoter to drive expression specifically in the liver. To note, we used a luciferase containing destabilizing sequences in the C-terminus of the protein as well as five consecutive AUUUA elements to the 3′ UTR to promote the protein and mRNA turnover, increasing the dynamic response of the signal (54). Mice were i.v. injected with the reporter AAVs, fed with high-fat diet (HFD) for 12 weeks to induce NAFLD and bioluminescence was quantified *in vivo*. No significant differences were observed between HFD-fed or control mice, suggesting that NAFLD is not associated with global changes in splicing efficiency (Figure 1D). While this result does not completely rule out potential changes in splicing efficiency, it suggests that the changes observed in Figure 1A are gene specific. Further supporting this hypothesis, global analysis of expression of 1 884 210 SINE elements in human NAFLD and control liver samples did not show any significant increase in exonization of intronic transposable elements associated with NAFLD (Supplementary Figure S1A).

We next used RNA-seq Quantification of Alternative Polyadenylation (QAPA) (51) to investigate potential changes in alternative mRNA polyadenylation. This analysis identified 1335 polyadenylation sites (dProximal Polyadenylation Usage (dPPAU) > 20) differentially utilized in NAFLD vs. control human liver samples. Principal component analysis confirmed the impact of NAFLD in mRNA polyadenylation (Figure 1E), increasing both distal and proximal alternative polyadenylation sites (Figure 1F). QAPA software specifically identifies alternative polyadenylation in terminal exons. To further investigate if differences in polyadenylation also affect intronic polyadenylation sites, we carried out a systematic analysis of genes involved in liver metabolism. This analysis revealed intronic polyadenylation events in key metabolic genes such as *PPARA*, *PPARD* and *NR1H4* (Figure 1G). Alignment to 3′ RNA-sequencing data using PolyA site atlas (59) further confirmed that these reads are consistent with dysregulated intronic polyadenylation that is exacerbated in NAFLD patients (Figure 1G bottom).

Altogether, these results show that early stages of human NAFLD are associated with defects in pre-mRNA processing and suggest that defects in polyadenylation could underly the dysregulation of key metabolic genes in the liver.

### PAR-CLIP analysis identifies the splicing factor SRSF10 as a potential regulator of mRNA polyadenylation

**Figure 2.**
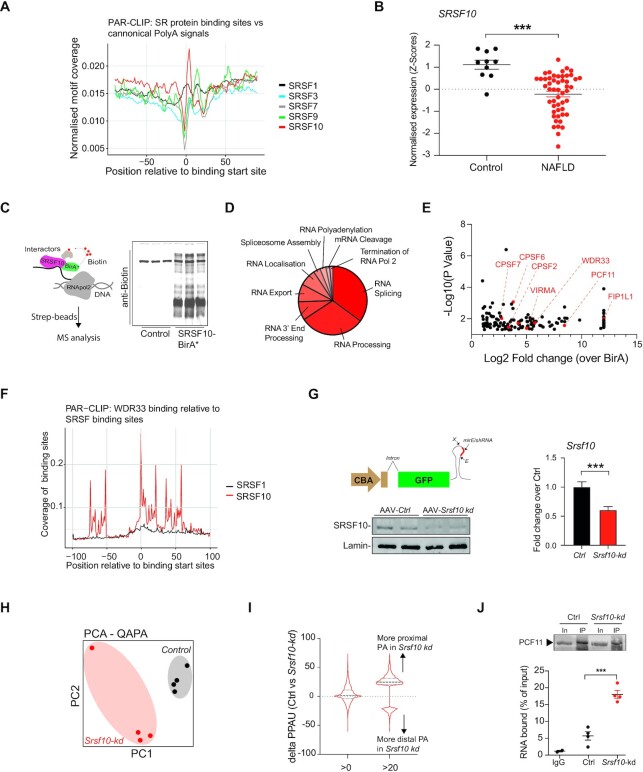
SRSF10 interacts with polyadenylation factors and directly binds prior to mRNA polyadenylation signals. (**A**) Analysis of PAR-CLIP data showing canonical polyadenylation site density relative to SR protein binding start sites. (**B**) Expression levels of *SRSF10* in the liver of control and NAFLD patients. *** *P*-value < 0.001. (**C**) Cartoon depicting proximity labelling approach to identify interacting proteins (left) and western blot analysis of biotinylation in cells transfected with SRSF10-BirA* versus Control-BirA* (*n* = 3). (**D**) Gene ontology analysis of SRSF10 interactors identified by proximity labelling. (**E**) Scatter plot of SRSF10 interactors with established roles in mRNA polyadenylation (highlighted in red). (**F**) Analysis of PAR-CLIP data showing WDR33 relative to SR protein binding start sites. (**G**) Cartoon depicting the design for the AAV backbone for simplified and efficient expression of an optimized mirE/shRNA in the liver (top); X-Xho1; E-EcoR1. Western blot analysis of SRSF10 from liver samples of *Srsf10-kd* or *Ctrl* mice (bottom). Left: qPCR analysis of *Srsf10* expression in liver samples of *Srsf10-kd* or *Ctrl* mice (*n* = 20). (**H**) Principal component analysis (PCA) plot of mRNA polyadenylation in liver from *Srsf10-kd* versus *Ctrl* mice fed a HFD. (**I**) Distribution of increased or decreased proximal polyadenylation usage with two different thresholds, >0 or >20 (dPPAU) upon SRSF10 inactivation in the liver. (**J**) Crosslinking and immunoprecipitation of PCF11 in the liver of *Srsf10-kd* or *Ctrl* mice. Top: western-blot showing PCF11 levels in the input and immuno-precipitate. Bottom: RNA crosslinked with PCF11 in the liver. Graphs show mean ± SEM. One-way ANOVA or a Mann–Whitney test was used (* *P*-value < 0.05, ** *P*-value < 0.01, *** *P*-value < 0.001).

To characterize the defects of pre-mRNA processing that accompany human NAFLD, we investigated whether the observed perturbations in hepatic polyadenylation profiles could be consequence of changes in the expression of RNA binding proteins. SR proteins have a long-established role in regulating RNA splicing and data suggests that they can play a role in alternative polyadenylation (25,45,60). In order to identify SR proteins which could play a role in both alternative splicing and alternative polyadenylation, a human photoactivatable ribonucleoside-enhanced crosslinking and immunoprecipitation (PAR-CLIP) dataset (61) was used to analyse genome-wide the binding proximity of five SR proteins to the consensus polyadenylation sequences (AAUAAA, AUUAAA, AAAUAA, AUAAAA and AUAAAU) (Figure 2A). PAR-CLIP binding analysis shows there is a strong enrichment of polyadenylation motifs immediately following SRSF10 binding sites, suggesting a potential role of this splicing factor in mRNA polyadenylation. Differential expression analysis of members of the SR and hnRNP RNA binding protein families in human liver samples showed that NAFLD is associated with a marked decrease in *SRSF10* expression (Figure 2B; Supplementary Figure S1B-C). Moreover, western-blot analysis showed that while other SR proteins such as SRSF1 and SRSF5 are unaffected in NAFLD in mouse liver, SRSF10 expression is markedly decreased (Supplementary Figure S2A). To further investigate this, we analysed the expression of SRSF10 in livers from mice fed two different obesogenic diets. This analysis confirmed that NAFLD progression is associated with decreased SRSF10 expression (Supplementary Figure S2B).

These results suggest that SRSF10 could play a previously uncharacterized role in controlling mRNA polyadenylation in the liver, and decreased SRSF10 expression could lead to dysregulated mRNA polyadenylation in NAFLD.

### SRSF10 regulates mRNA polyadenylation in the liver

While SRSF10 is a well-established regulator of AS, a role in alternative polyadenylation has not been reported (62). To further investigate such a potential function of SRSF10 we performed a proximity labelling (BioID) interactome analysis in HeLa cells by expressing SRSF10 fused to a promiscuous biotin ligase (BirA) (63)(Figure 2C, left). Expression of SRSF10-BirA constructs was confirmed by western blot analysis (Supplementary Figure S2C) and biotinylated interactors were captured by affinity purification and identified by mass spectrometry (MS) (Figure 2C, right). The full list of interacting proteins is provided in Supplementary Table S1. Gene ontology analysis shows that SRSF10 interacts with factors from the spliceosome and polyadenylation machinery as well as those involved in nuclear export (Figure 2D). Notably, 7 bona fide polyadenylation factors (28) including four members of the cleavage and polyadenylation specificity factor complex (CPSF2, 6 and 7 and FIP1L1), the M6A methyltransferase VIRMA, the pre-mRNA 3′ end processing protein WDR33 and the cleavage and polyadenylation factor subunit PCF11, were identified as SRSF10 interactors (Figure 2E), further suggesting a potential role for SRSF10 in mRNA polyadenylation. To test this hypothesis, we performed a comparison of a human PAR-CLIP dataset for WDR33 (64) with the previously used SRSF10 PAR-CLIP dataset. This analysis shows highly enriched WDR33 binding in the region surrounding SRSF10 binding sites as well as directly overlapping binding sites (Figure 2F) suggesting close proximity of binding and even potential competition between polyadenylation factors and SRSF10 for mRNA binding.

To directly test this hypothesis in a pathophysiological context *in vivo*, we generated a loss-of-function model for *Srsf10* in the liver and induced obesity using a high fat diet. To this end, we first engineered a novel AAV vector named (*AAV-CBA-mirE/shRNA*) for the liver-specific delivery of a mirE/shRNA (Figure 2G; left, top), by incorporating in the AAV2/8 genome the optimized miR-30 backbone (mirE). This approach has been previously developed in lenti- and retro-viruses, and increases mature shRNA levels and knockdown efficiency (53). Mice injected with an AAV-CBA-mirE/shRNA to *Srsf10* (AAV-*Srsf10-kd*) showed a significant decrease in SRSF10 levels in the liver at both protein (Figure 2G; left, bottom) and RNA levels (65) (Figure 2G, right) compared with mice expressing a control mirE/shRNA (AAV-*Ctrl*).

Genome-wide mRNA polyadenylation analysis by RNA-seq demonstrates a strong effect of SRSF10 deficiency in mRNA polyadenylation events (Figure 2H). Additional analysis of differential polyadenylation events (dPPAU > 20) revealed that SRSF10 deficiency promotes preferential use of proximal polyadenylation sites (Figure 2I). To experimentally investigate the molecular basis of this effect we performed a crosslinking and immunoprecipitation of PCF11, one of the main polyadenylation factors interacting with SRSF10 (Figure 2E), in *Srsf10-kd* versus *Ctrl* livers. This analysis showed that inactivation of SRSF10 in the liver is associated with the increase in RNA binding of PCF11, suggesting that SRSF10 prevents the interaction of the mRNA polyadenylation machinery with intronic polyadenylation sites (Figure 2J).

Collectively, these results show that SRSF10 interacts with and binds in close proximity to polyadenylation factors and its deficiency leads to increased RNA binding of specific components of the polyadenylation machinery and the use of proximal polyadenylation sites. In association with the previous PAR-CLIP analysis, mechanistically this shows that SRSF10 binds RNA adjacent to polyadenylation signals and prevents the binding of polyadenylation factors promoting the use of distal polyadenylation sites.

### Inactivation of SRSF10 downregulates PPARα signalling and exacerbates NAFLD and metabolic dysfunction

**Figure 3.**
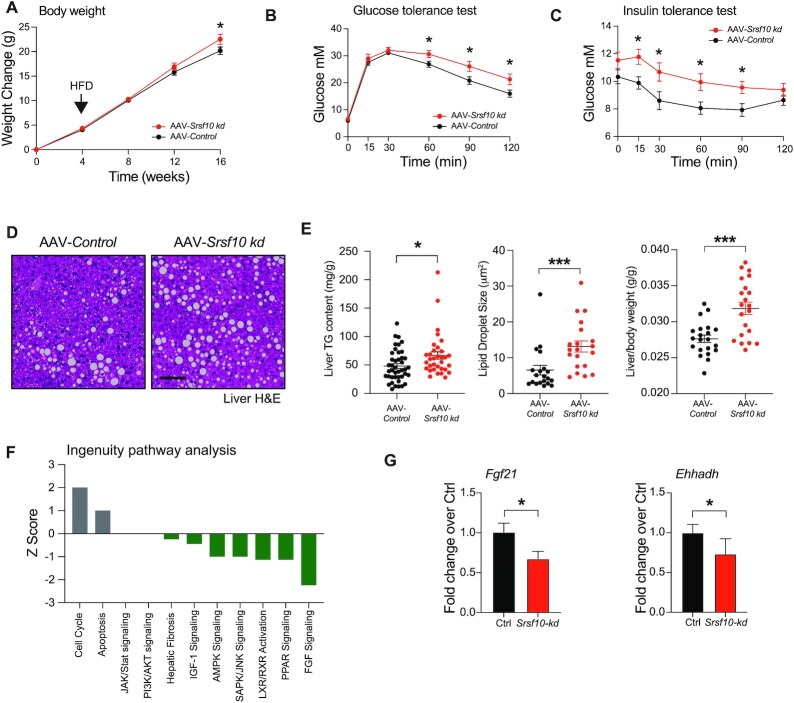
SRSF10 inactivation promotes obesity-induced metabolic dysfunction. (**A**) Body-weight change over time (*n* = 28–34). (**B**) Glucose (1 g/kg) and (**C**) Insulin tolerance tests performed after 12 weeks of HFD (*n* = 11–12). (**D**) Representative H&E-stained liver samples. Scale bar: 100 μm. (**E**) Quantification of liver triglyceride content (left) (*n* = 33–42), droplet size using ImageJ (middle) and liver/body weight ratio (right) (*n* = 21). (**F**) Ingenuity pathway analysis of RNA-seq differential expression in the liver of *Srsf10-kd* and *Ctrl* mice (*n* = 4). (**G**) qPCR analysis of known PPARα target genes, *Fgf21* and *Ehhadh*. Graphs show mean ± SEM. Two-way ANOVA or a Mann–Whitney test was used (* *P*-value < 0.05, ** *P*-value < 0.01, *** *P*-value < 0.001).

Mice injected with AAV-*Srsf10-kd* showed no changes to body weight or glucose and insulin tolerance under control diet conditions. However, when provided with a high fat diet, AAV-*Srsf10-kd* mice show a mild increase in body weight (Figure 3A). Further characterization revealed that SRSF10 inactivation is associated with marked impairment of glucose tolerance (Figure 3B) and insulin sensitivity (Figure 3C). Histologically, *Srsf10-kd* mice showed increased steatosis as determined by H&E (Figure 3D) and direct quantification of intrahepatic triglyceride content (Figure 3E). Moreover, increased steatosis was associated with increased lipid droplet size and liver/body weight ratio (Figure 3E).

To investigate the molecular mechanisms underlying this phenotype, we performed the Ingenuity pathway analysis of RNA-seq from the liver of *Srsf10-kd* and *Ctrl* mice. Differentially expressed genes showed a downregulation of key metabolic pathways such as FGF signalling or PPAR signalling (Figure 3F). Comparison of a microarray from livers following *Ppara* gene knock-out (encoding PPARα) and the RNA-seq presented here shows that gene expression changes associated with SRSF10 inactivation is positively correlated with changes associated with PPARα inactivation (*R*^2^ = 0.104; *P* value = 0.00107; Pearson correlation coefficient) (Supplementary Figure S2D). Direct RNA-seq analysis confirmed that inactivation of SRSF10 is associated with decreased expression of genes induced by PPARα such as *Lpin2* and increased expression of genes repressed by PPARα such as *Nfkbia* (66) (Supplementary Figure S2E). Notably, *Ppara* was significantly downregulated upon SRSF10 inactivation (Supplementary Figure S2E). Validation by qPCR analysis in an additional set of samples confirmed a significant decrease in metabolically relevant genes such as the hepatokine *Fgf21* and lipolysis factor *Ehhadh*, two well characterized PPARα targets (Figure 3G) (67).

Liver PPARα signalling has previously been described to regulate whole body metabolism in part by regulating lipid accumulation in white and brown adipose tissue, a mechanism largely mediated by FGF21 (67). Given the observed changes in body-weight gain and *Fgf21* expression in the liver, analysis of adipose tissue was undertaken. Brown adipose tissue from *Srsf10*-*kd* showed a significant shift towards increased whitening (Supplementary Figure S3A) and increased unilocular lipid droplets (Supplementary Figure S3B). Consistently, expression of the brown adipose tissue marker *Ucp-1* was reduced in *Srsf10-kd* mice (Supplementary Figure S3C).

These results show that loss-of-function of SRSF10 in the liver is associated with decreased PPARα activity leading to increased susceptibility to NAFLD and obesity-induced metabolic dysfunction.

### SRSF10 prevents intronic polyadenylation in obesity-induced liver disease

As previously shown, we observed a strong shift towards proximal polyadenylation sites together with decreased polyadenylation factor binding in *Srsf10* deficient livers. Following the observed dysregulation of PPARα signalling in *Srsf10*-deficient mice we hypothesized that decreased SRSF10 could contribute to metabolic dysfunction by dysregulating mRNA polyadenylation and hence PPARα target gene expression. To test this hypothesis and further elucidate the molecular mechanisms underlying the observed phenotype, we performed a direct analysis of intronic polyadenylation events with standard RNA-seq and IPA finder (68).

**Figure 4.**
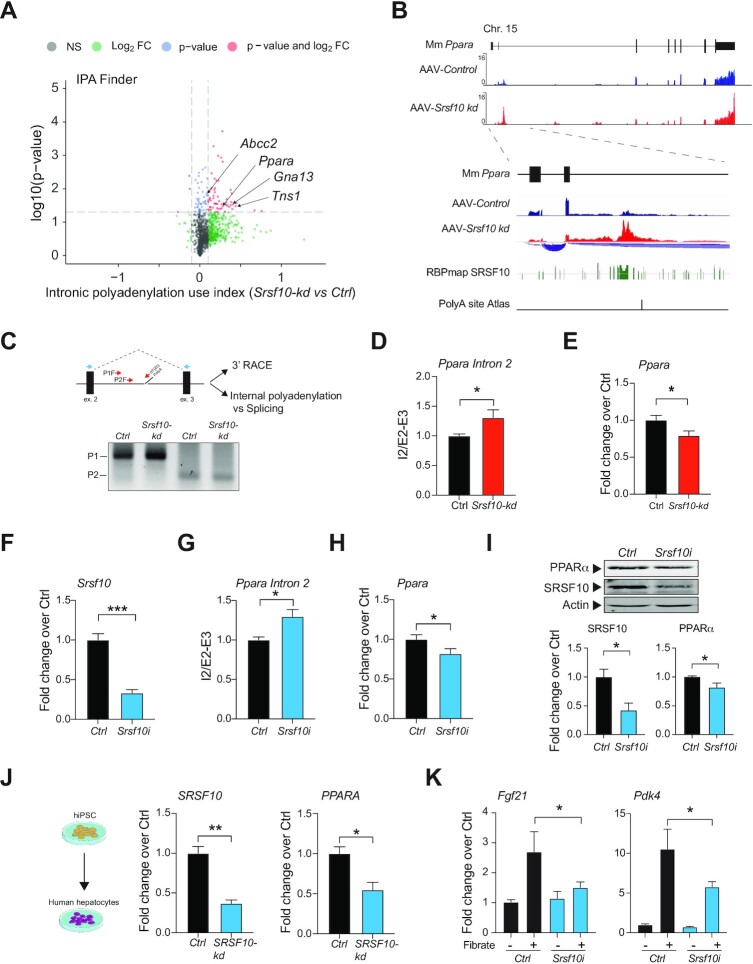
Inactivation of SRSF10 is associated with dysregulation of mRNA polyadenylation in the liver. (**A**) Intronic polyadenylation analysis from *Srsf10-kd* and *Ctrl* liver samples analysed by IPA finder. (**B**) RNA-seq tracks from *Srsf10-kd* and *Ctrl* liver samples aligned with refseq layout of the *Ppara* gene, sashimi plot showing the splicing patterns. RBPmap predicted binding sites for SRSF10 are indicated in green lines and bottom track shows validated 3′ RNA sequencing data indicating potential intronic polyadenylation sites. (**C**) 3′RACE PCR analysed on an agarose gel using two different gene specific forward primers (P1F and P2F). (**D**) qPCR analysis of intron 2 abundance in *Srsf10-kd* and *Ctrl* liver samples using primers in the intron proximal to the 5′ splice site and primers in exons 2 and 3. (**E**) qPCR analysis of *Ppara* gene expression in liver samples of *Srsf10-kd* or *Ctrl* mice. (**F**) qPCR analysis of *Srsf10* expression following transfection of siRNA to *Srsf10* or control in mouse primary hepatocytes. (**G**) qPCR analysis of expression of *Ppara* intron 2 relative to expression of exon 2 to exon 3 spliced RNA (*n* = 6) and (**H**) qPCR analysis of *Ppara* expression. (**I**) Western blot analysis of SRSF10 and PPARα in hepatocytes after *Srsf10* silencing (*n* = 4). (**J**) qPCR analysis of *SRSF10* and *PPARA* expression in human hepatocytes upon lentivirus-mediated *SRSF10* knock-down. (**K**) qPCR analysis of *Fgf21* and *Pdk4* expression following siRNA treatment and subsequent treatment of fenofibrate for 4 or 16 h (*n* = 13–20). Graphs show mean ± SEM (*n* = 20–25 unless indicated otherwise); One-way anova, Mann–Whitney or Students t-test were used for statistical comparison (* *P*-value < 0.05, ** *P*-value < 0.01, *** *P*-value < 0.001).

This analysis revealed an overall increase in intronic polyadenylation events in *Srsf10-kd* livers, affecting genes such as *Abcc2*, *Tns1*, *Gna13*, *Acsl3* or *Klf10* (Figure 4A; Supplementary Figure S4A–E), consistent with the role for SRSF10 in the regulation of polyadenylation of NAFLD-relevant genes. Notably, *Ppara*, was identified among the top genes with increased intronic polyadenylation associated with SRSF10 inactivation (Figure 4A). An obvious candidate to explain the decreased PPARα signalling in the liver of *Srsf10-kd* mice is *Ppara* gene itself. To confirm this idea we performed a direct analysis of this region, revealing previously described characteristics of intronic polyadenylation and premature transcription termination, including elevated reads at the 3′ end, close proximity to the 5′ end of the transcript, location within a large intron of a transcription factor gene and leading to a modest decrease in the expression of the full gene (44,69,70). Additionally, strong polyA signals from 3′ RNA-sequencing data (59) further confirmed that these reads are consistent with dysregulated intronic polyadenylation that is exacerbated upon SRSF10 inactivation (Figure 4B, bottom). Analysis of potential binding sites for SRSF10 revealed the proximity of these signals, consistent with a role in repressing a cryptic polyadenylation signal. In order to confirm the presence of a novel polyadenylated transcript, 3′ rapid amplification of cDNA ends (RACE) was performed. 3′RACE and subsequent PCR analysis using two different sets of primers gave bands of the expected size, consistent with alternative polyadenylation products that was further verified by subsequent Sanger sequencing (sequence provided in Supplementary sequence RACE), confirming a role for SRSF10 repressing this cryptic polyadenylation signal (Figure 4C). This effect was further confirmed and quantified in *Srsf10-kd* versus *Ctrl* liver samples by qPCR analysis of the intronic polyadenylation fragment compared to the spliced transcript (Figure 4D). Moreover, the increase in alternative polyadenylation was associated with decreased *Ppara* expression (Figure 4E, Supplementary Figure S2E), and decreased PPARα signalling (Figure 3F, G).

Finally, other potential mechanisms such as cryptic exon usage, alternative splice site usage within the primary transcript or potential antisense transcripts, were ruled out by PCR and RNA-seq splicing analysis (Figure 4B, blue sashimi plot) and analysis of antisense mapped reads (Supplementary Figure S4F) respectively.

These results show that SRSF10 is a key regulator of intronic polyadenylation in the liver and its downregulation in NAFLD contributes to impaired polyadenylation and decreased expression of *Ppara*.

### SRSF10 prevents intronic PPARA polyadenylation in mouse and human hepatocytes

To confirm the effect of SRSF10 on mRNA polyadenylation and PPARα signalling in a cell-autonomous system, mouse primary hepatocytes were isolated and transfected with an siRNA against *Srsf10*. Efficient inactivation of *Srsf10* (>70% after 48 h) was first confirmed at the mRNA level (Figure 4F). Consistent with the results obtained *in vivo*, inactivation of *Srsf10* is associated with increased intronic polyadenylation (Figure 4G) and decreased expression (Figure 4H) of *Ppara* gene in mouse hepatocytes. These results were confirmed at the protein level by western blot analysis (Figure 4I). Moreover, human iPSC-derived hepatocytes were infected with a lentivirus expressing a mirE/shRNA against *SRSF10*. Highly efficient transduction of human hepatocytes was evaluated using a GFP reporter (Supplementary Figure S5A). An additional analysis showed that *SRSF10* inactivation was associated with the downregulation of *PPARA* expression (Figure 4J and Supplementary Figure S5B), confirming that the effect of SRSF10 is conserved in human hepatocytes. To further validate the effect of SRSF10 in controlling PPARα signalling, primary hepatocytes were transfected with siRNA to *Srsf10* or siRNA control and treated with the PPARα agonist fenofibrate or control vehicle. qPCR analysis showed that SRSF10 inactivation impairs the expression of *Fgf2*1 and *Pdk4*, two canonical PPARα-responsive genes (67) further confirming the functional effect of SRSF10 in PPARα signalling (Figure 4K).

These results collectively show that SRSF10 is involved in the repression of intronic polyadenylation sites, and inactivation of SRSF10 in hepatocytes leads to increased intronic polyadenylation of the *Ppara* transcript and decreased PPARα signalling.

## DISCUSSION

RNA processing plays a central role in the regulation of gene expression. Consequently, defects in core RNA processing mechanisms have been implicated in a growing number of human pathologies. In particular, alterations in RNA polyadenylation due to mutations in canonical polyadenylation signals are associated with diverse pathologies including systemic lupus erythematosus (71,72) or thalassaemia (73). Moreover, genome wide perturbations in RNA polyadenylation have been associated with the majority of cancer types (74–77). However, the contribution of RNA polyadenylation to liver disease has not been studied. Here we show that mishandling of mRNA maturation is a major hallmark and potential driver for liver disease. We find that while constitutive mRNA splicing remains active, mRNA polyadenylation is significantly affected in the liver of NAFLD patients. Moreover, by combining *in vivo* and *in vitro* studies, we uncover a novel role for SRSF10 in controlling mRNA polyadenylation in mouse and human hepatocytes.

Mechanistically, we show that SRSF10 interacts with and binds in close proximity to a number of the polyadenylation factors such as WDR33 and PCF11. Furthermore, SRSF10 binding was found to be immediately prior to canonical polyadenylation sequences, supporting the idea that SRSF10 represses the recognition and use of cryptic intronic polyadenylation sites. This is the first evidence for an SR protein influencing sequence-dependent RNA intronic polyadenylation.

**Figure 5.**
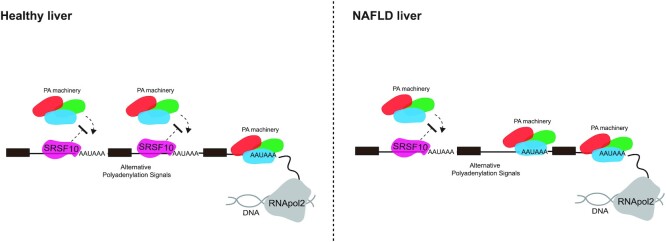
Cartoon depicting a potential model for the role of SRSF10 in mRNA polyadenylation. SRSF10 interacts with and binds in close proximity to a number of the polyadenylation factors and can prevent their binding to RNA, inhibiting cryptic intronic polyadenylation.

Interestingly, SRSF10 is a known U1 snRNP interactor (78) and this interaction is supported by our interactome data. Beyond its central role in controlling intron splicing, evidence show that U1 snRNP telescripting plays a key role in preventing premature polyadenylation and promoting long-range transcriptional elongation (23,24,79–81). Whilst the exact mode of action for how SRSF10 inhibits gene-specific aberrant polyadenylation and the contribution of the SRSF10-U1snRNP interaction in this process require further studies, our data suggest that SRSF10 could assist in telescripting by competing for binding sites surrounding polyadenylation signals and inhibiting the recruitment of polyadenylation factors such as PCF11, a known mediator of intronic polyadenylation (Figure 5).

Consistent with this new role for SRSF10, its inactivation in human and mouse hepatocytes, leads to intronic polyadenylation and decreased expression of key metabolic genes such as the nuclear receptor PPARα. In addition, liver-specific knockdown of *Srsf10* leads to increased cryptic intronic polyadenylation and decreased expression of PPARα in the liver. These transcriptional perturbations are associated with exacerbated NAFLD, together with an increase in body-weight gain, increased insulin resistance and glucose intolerance under obesogenic conditions. We found that SRSF10 expression is decreased in the liver of human patients and mouse models of NAFLD associated with obesogenic diets. Notably, the *SRSF10* locus has been recently associated with type-2 diabetes adjusted for body mass index in east Asian individuals (82), but the molecular basis of this association has not been established. Our results shed light on the role of SRSF10 in metabolic regulation in health and disease in the liver.

SRSF10 is a well-established regulator of pre-mRNA alternative splicing and constitutive splicing, however no role in RNA polyadenylation had been described before. Similar to the dual role for SRSF10 in splicing, being both a sequence-dependant regulator and general repressor depending on its phosphorylation status (41,46,83–85), a dual role in controlling mRNA polyadenylation depending on post-translational modifications could be predicted. While this warrants further studies, our work implicates dysregulated intronic polyadenylation in the metabolic dysfunction associated with NAFLD, and uncovers a key role for SRSF10 in this process.

## DATA AVAILABILITY

Bulk RNA-seq data generated for this study have been deposited at GEO under accession number GSE179964. The mass spectrometry proteomics data have been deposited to the ProteomeXchange Consortium via the PRIDE partner repository with the dataset identifier PXD029228.

## Supplementary Material

gkac165_Supplemental_Files
