# Supplementary material for: Dysregulated RNA polyadenylation contributes to metabolic impairment in non-alcoholic fatty liver disease
Source: Nucleic Acids Res. 2022 Mar 16;50(6):3379–93. doi: 10.1093/nar/gkac165 (PMC8989518; doi:10.1093/nar/gkac165)
Supplement: gkac165_Supplemental_Files [file gkac165_supplemental_files.zip › Fig Suppl S1 to S5.pdf]

Figure S1

Jobbins et al.

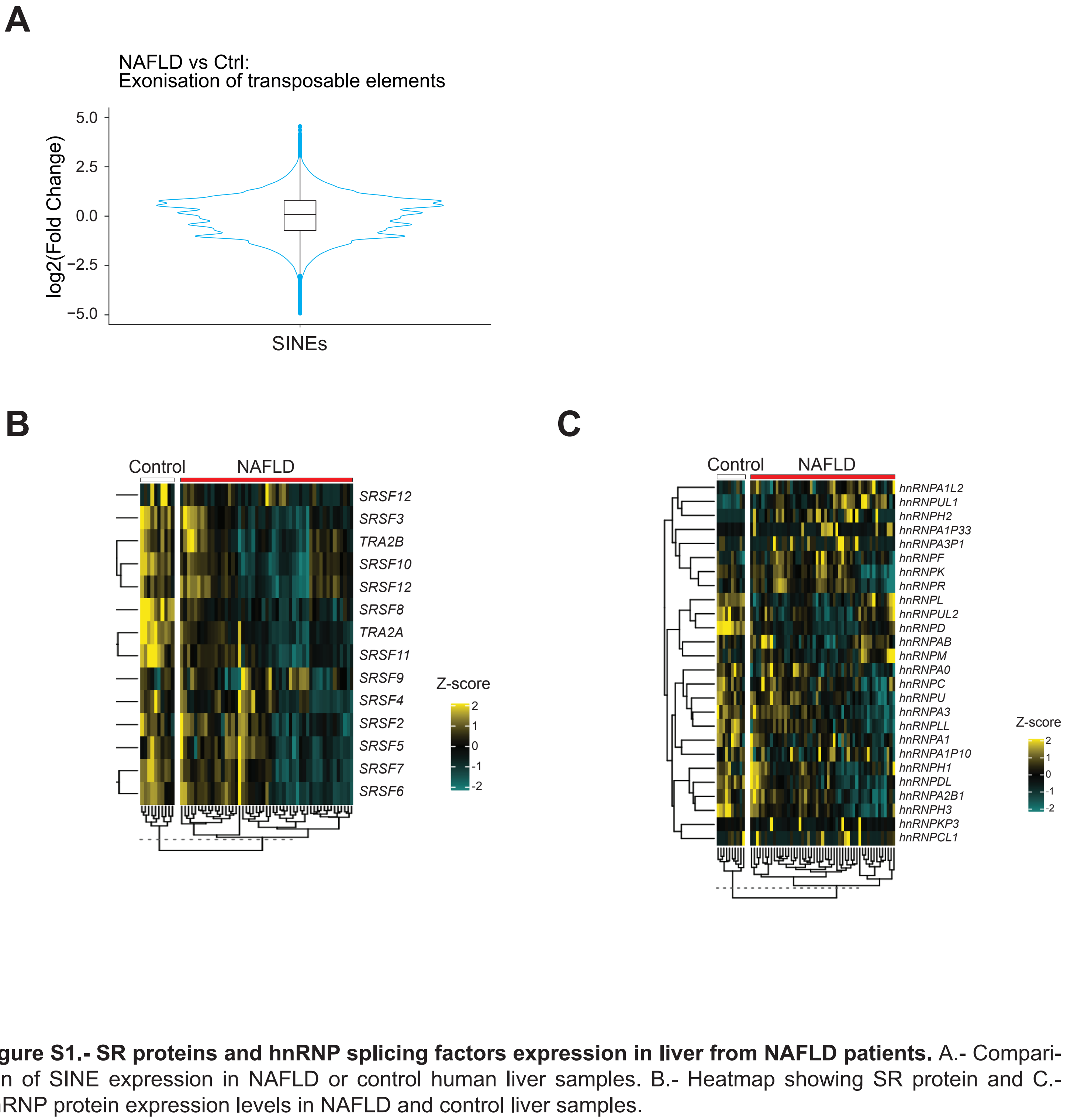

Figure S1.- SR proteins and hnRNP splicing factors expression in liver from NAFLD patients. A.- Comparison of SINE expression in NAFLD or control human liver samples. B.- Heatmap showing SR protein and C.- hnRNP protein expression levels in NAFLD and control liver samples.

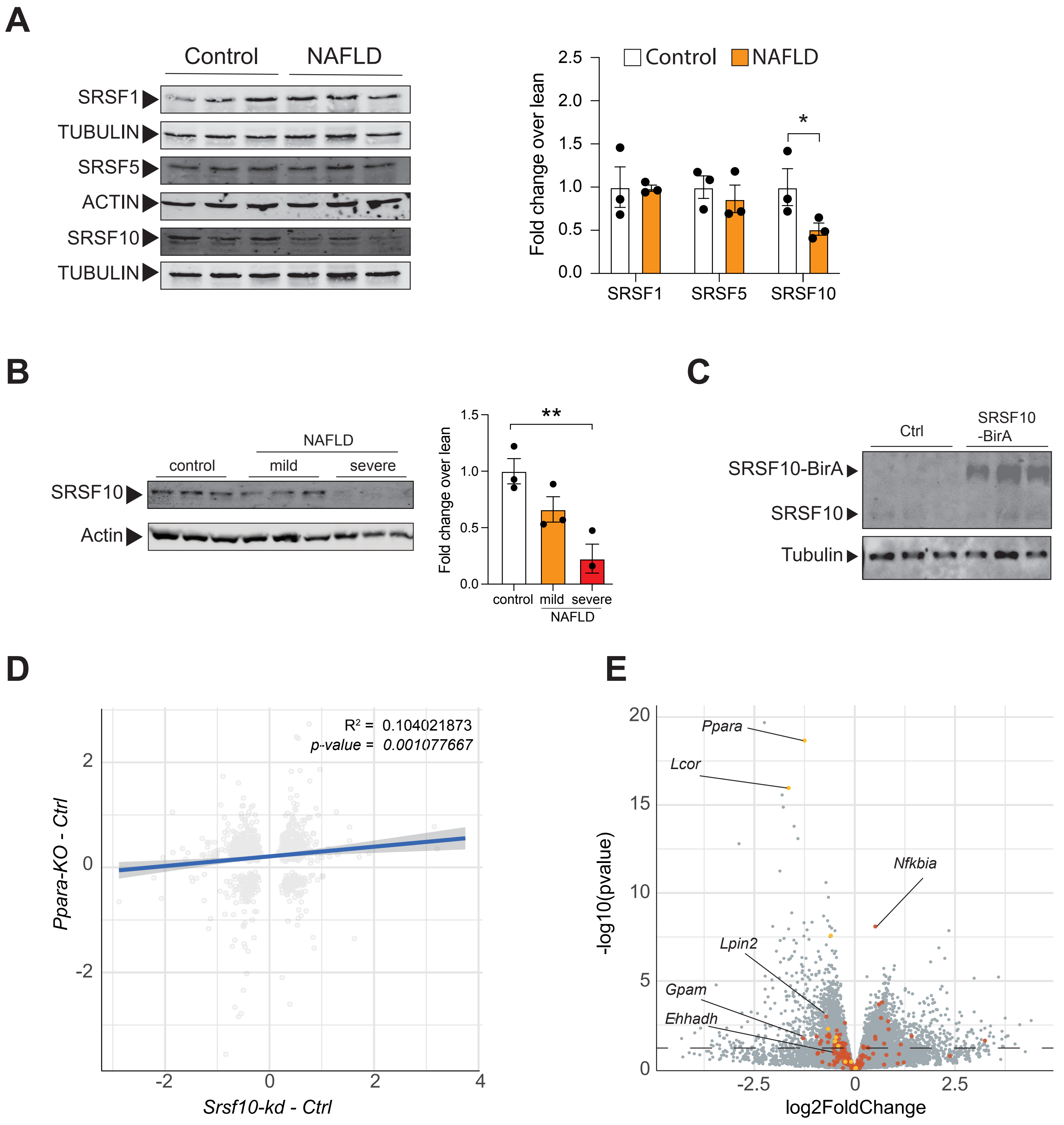

Figure S2.- SRSF10 protein levels are decreased in NAFLD models in mice. A.- Western blot analysis showing SRSF1, SRSF5 and SRSF10 from liver samples taken from mice fed a control diet, and to obesogenic diets to promote NAFLD B.- Western-blot analysis showing the expression of SRSF10 in liver from mice with mild (fed a high fat diet) or severe (fed a western diet) NAFLD (n=3). C.- Western blot analysis of SRSF10 and SRSF10-BioID expression.D.- Comparision of differentially expressed genes in *Ppara* knock out and *Srsf10-kd* livers . E.- Expression of PPARα target genes (orange) and PPARα signalling regulators (yellow) in liver from *Srsf10-KD* livers. Graphs show mean±SEM. Students t-test was used for statistical comparison (* p-value<0.05; ** p-value<0.01).

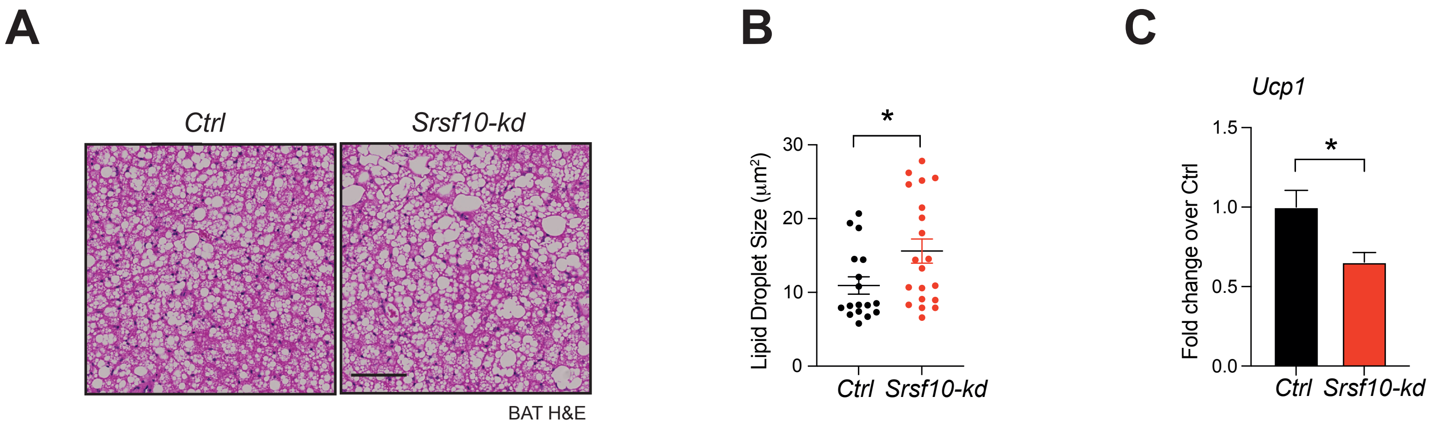

Figure S3.- Impaired PPARα signalling in liver leads to altered brown adipose tissue morphology and downregulation of *Ucp1*. A.- Representative H&E staining of brown adipose tissue samples. B.- Quantification of lipid droplet size using ImageJ. C.- qPCR analysis of *Ucp1* expression in brown adipose tissue. Graphs show mean±SEM; n=20. Mann-Whitney test was used for statistical comparison (* p-value<0.05).

Figure S4

Jobbins et al.

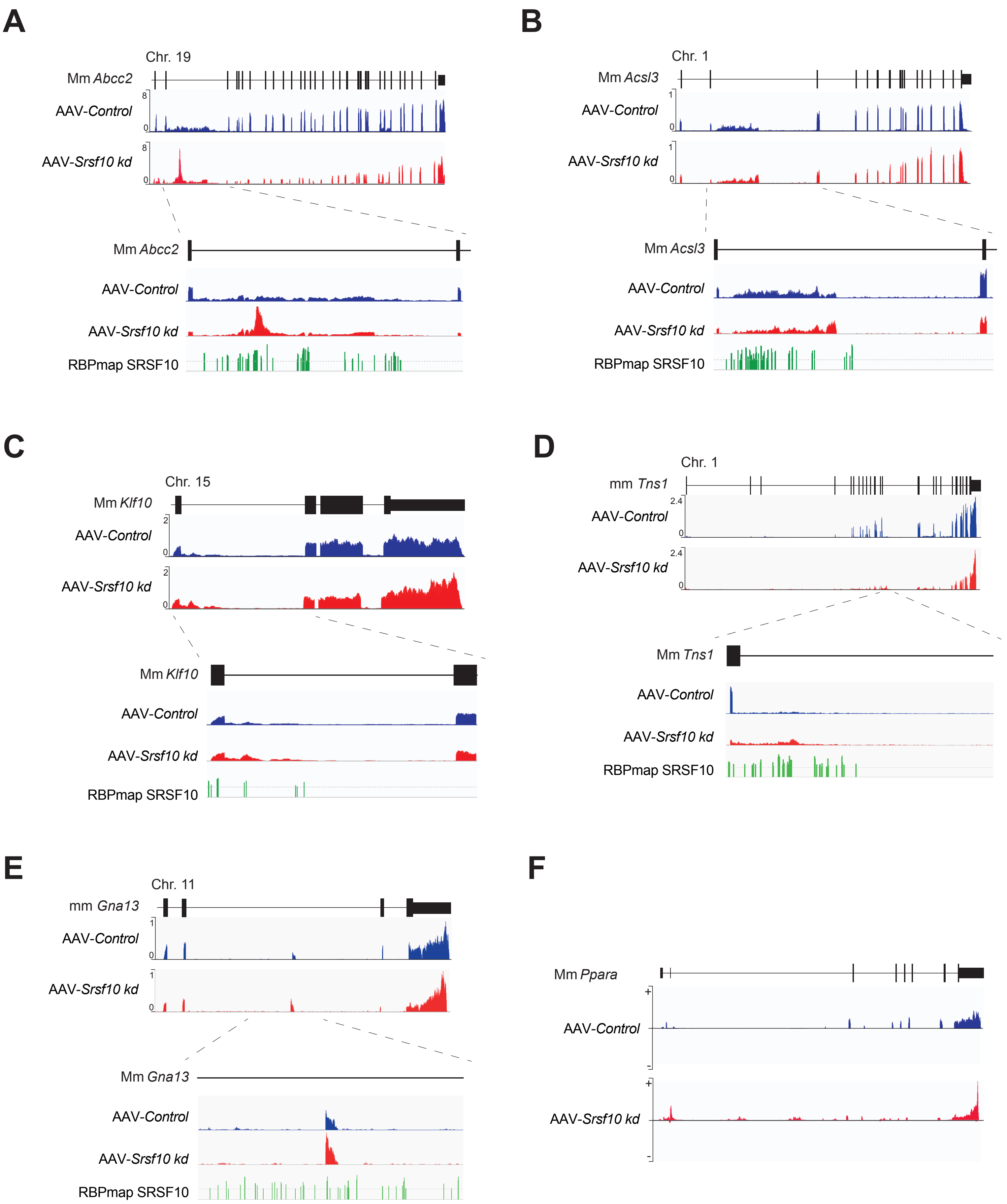

Figure S4.- Inactivation of PPARα leads to increased intronic polyadenylation in the liver.
A.- RNA-seq tracks for *Srsf10-kd* and *Control* mice for exemplar genes showing intronic polyadenylation. Refseq gene layout and RBPmap predicted SRSF10 binding sites for *Abcc2*, *Acsl3* (B), *Klf10* (C), *Tns1* (D) and *Gna13* (E).
F.- RNA-seq tracks for Ppara split by sense (+) and antisense (-) direction.

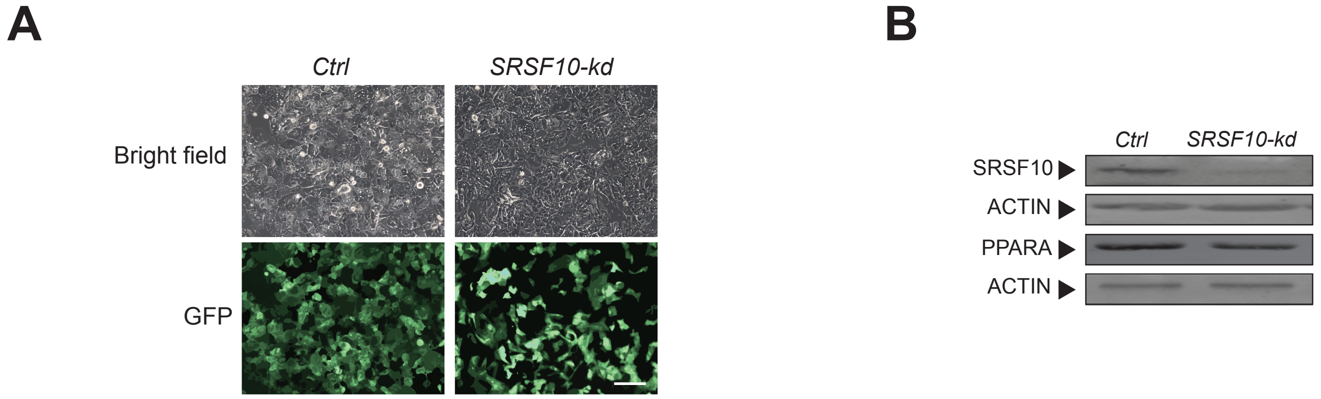

Figure S5.- iPSC-derived human hepatocytes are efficiently transduced with lentiviruses expressing mirE/shRNA to SRSF10. A.- Brightfield (top) and GFP (bottom) images of human iPSC-derived hepatocytes following lentiviral infection with a control vector expressing a mirE/shRNA to luciferase or *SRSF10*. B.- Western blot analysis of SRSF10 and PPARα expression.
