# Supplementary material for: Dysregulated RNA polyadenylation contributes to metabolic impairment in non-alcoholic fatty liver disease
Source: Nucleic Acids Res. 2022 Mar 16;50(6):3379–93. doi: 10.1093/nar/gkac165 (PMC8989518; doi:10.1093/nar/gkac165)
Supplement: gkac165_Supplemental_Files [file gkac165_supplemental_files.zip › Supplementary sequence RACE.docx]

**Sequence obtained from sanger sequencing of 3’ RACE PCR product:**

gccaggctacatggaacctggtctcaaaaacaaatcaatagccaggcacagtgatacgttcttttaatccttatactcaggagacagaggcaggaggttctctatgagttcaagtccagcttggtctacacacaga
